# Use of a real-size 3D-printed model as a preoperative and intraoperative tool for minimally invasive plating of comminuted midshaft clavicle fractures

**DOI:** 10.1186/s13018-015-0233-5

**Published:** 2015-06-10

**Authors:** Hyong Nyun Kim, Xiao Ning Liu, Kyu Cheol Noh

**Affiliations:** Department of Orthopaedic Surgery, Kangnam Sacred Heart Hospital, Hallym University College of Medicine, 948-1, Dalim-1dong, Youngdeungpo-gu, Seoul, 150-950 South Korea; Department of Orthopaedic Surgery, The Second Hospital, Jilin University, Changchun, China

**Keywords:** 3D printing, Clavicle fracture, Minimally invasive plate osteosynthesis, Precontoured plate

## Abstract

**Background:**

Open reduction and plate fixation is the standard operative treatment for displaced midshaft clavicle fracture. However, sometimes it is difficult to achieve anatomic reduction by open reduction technique in cases with comminution.

**Methods:**

We describe a novel technique using a real-size three dimensionally (3D)-printed clavicle model as a preoperative and intraoperative tool for minimally invasive plating of displaced comminuted midshaft clavicle fractures. A computed tomography (CT) scan is taken of both clavicles in patients with a unilateral displaced comminuted midshaft clavicle fracture. Both clavicles are 3D printed into a real-size clavicle model. Using the mirror imaging technique, the uninjured side clavicle is 3D printed into the opposite side model to produce a suitable replica of the fractured side clavicle pre-injury.

**Results:**

The 3D-printed fractured clavicle model allows the surgeon to observe and manipulate accurate anatomical replicas of the fractured bone to assist in fracture reduction prior to surgery. The 3D-printed uninjured clavicle model can be utilized as a template to select the anatomically precontoured locking plate which best fits the model. The plate can be inserted through a small incision and fixed with locking screws without exposing the fracture site. Seven comminuted clavicle fractures treated with this technique achieved good bone union.

**Conclusions:**

This technique can be used for a unilateral displaced comminuted midshaft clavicle fracture when it is difficult to achieve anatomic reduction by open reduction technique.

*Level of evidence* V.

## Background

Open reduction and plate fixation is the standard operative treatment for displaced midshaft clavicle fracture [1]. This procedure provides biomechanically stable construction allowing for early mobilization and the accommodation of fracture compression. However, extensive stripping of the fracture site results in an increased risk of infection and a potential decrease in cosmetic satisfaction. Complications associated with plate fixation, such as implant failure or re-fracture after implant removal, have been reported [2, 3]. The elastic intramedullary nailing technique was developed to preserve the periosteal blood supply of the fracture area. It has the advantage of maintaining an intact fracture hematoma, which may increase the rate of fracture healing [4–7]. However, an intramedullary device may migrate with the movements of the bone and can therefore result in medial protrusion, thus causing irritation or skin perforation. Telescoping and shortening are common in comminuted fractures treated with elastic intramedullary nailing, and early motion is limited due to its suboptimal stability [7].

A minimally invasive plate osteosynthesis (MIPO) has been introduced to overcome the limitations of the two commonly used operative techniques, as it can provide excellent biological healing and optimal stabilization strength [1, 8–11]. However, it is more technically demanding than standard open reduction and plate fixation as the fracture site remains closed. There are numerous manufacturers that supply anatomically designed precontoured locking plates for use in the minimally invasive approach. However, it is challenging to select the right plate for each clavicle fracture due to the bones’ individual size and shape characteristics. It is difficult to check if the plate fits well on the clavicle unless the clavicle is fully exposed and the plate is inserted into position. If the plate does not attach suitably to the fractured clavicle, adequate screw fixation for optimal stability may not be possible. This may cause the plate to protrude and therefore lead to skin irritation. A plate can be molded to fit the clavicle, but this process is difficult if the clavicle is not fully exposed.

In this article, we describe a novel technique using a real-size three dimensionally (3D)-printed clavicle model. The 3D-printed clavicle is utilized as a template to select the anatomically precontoured locking plate for the minimally invasive approach for a superior fit to the clavicle prior to surgery. A computed tomography (CT) scan is taken of both clavicles in patients with a unilateral displaced comminuted midshaft clavicle fracture. Using the mirror imaging technique, the contralateral uninjured clavicle is printed into a real-size model of the fractured side clavicle. This replica is employed as a template and utilized intraoperatively as a reference for anatomic reduction of the fracture.

## Methods

### Indications

This technique is indicated for a unilateral displaced comminuted midshaft clavicle fracture that requires operative treatment. Comminuted fractures that are difficult to achieve anatomic reduction by open reduction technique may benefit the most with this process. Fractures with poor soft tissue condition that may lead to wound problems after opening the fracture site may benefit with this minimally invasive technique without injuring the compromised soft tissue. The contralateral uninjured clavicle should not present with fracture, deformity, or history of surgery to be deemed suitable as an anatomically precontoured locking plate template. The template will be utilized as a reference for anatomic reduction during the operation.

### Model production by 3D printing

**Fig. 1:**
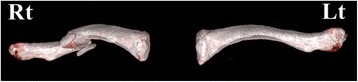
A CT scan is taken of both clavicles, with a slice thickness of 1 mm

CT scan is a data acquisition tool for 3D printing. A CT scan is taken of both clavicles with a slice thickness of 1 mm (Fig. 1). The data acquired from the CT scan is saved in the DICOM (Digital Imaging and Communications in Medicine) format. Surgeons can either use commercial companies offering 3D printing services or use a 3D printing machine to produce a real-size 3D-printed model. An increasing number of commercial companies are offering 3D printing services. We chose to outsource the 3D printing requirements to commercial companies as they are more efficient at converting the DICOM data to a STL (Standard Triangulation Language) file format that is utilized by the 3D printing machine.

**Fig. 2:**
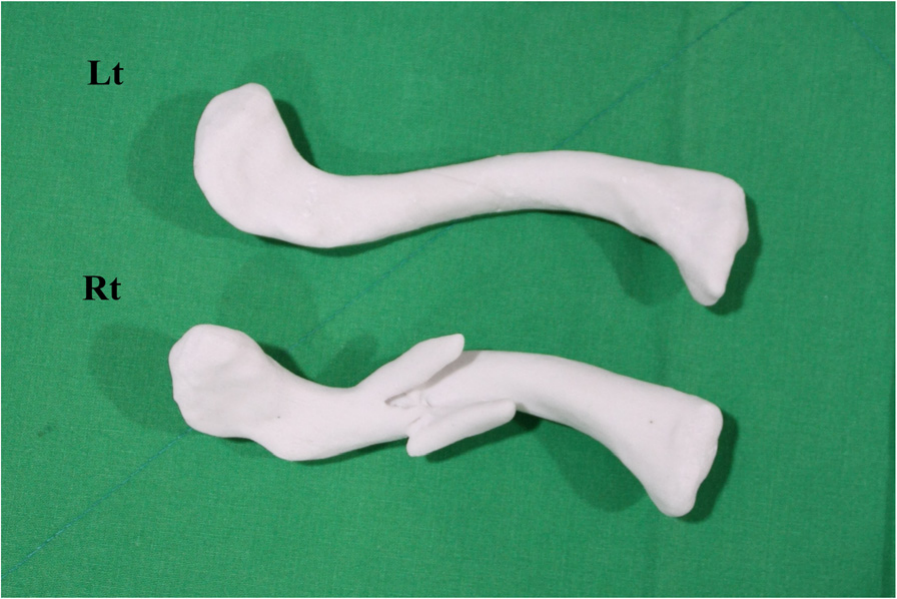
A real-size fractured clavicle model is 3D printed (*Rt*). Using the mirror imaging technique, the undamaged left side clavicle model is also printed to become a suitable replica of the fractured right side clavicle prior to injury (*Lt*)

Under the patient's agreement to use the data and the agreement with the 3D printing company to safely protect the data and destroy it upon completion of the model, the data (in DICOM format) is sent to a 3D printing company to produce a real-size clavicle model. The 3D printing company converts the DICOM data into a STL file format using specialized software called MIMICS (Materialise Interactive Medical Image Control System Software, Materialise, Belgium). A real-size fractured clavicle model is 3D-printed utilizing an inkjet printing technique via a 3D printing machine (Projet x60 series, 3D System Inc., Rock Hill, SC, USA). Using the mirror imaging technique, the uninjured side clavicle is 3D-printed to produce a suitable replica of the damaged side clavicle pre-injury (Fig. 2). To minimize the overall cost of model production, attention was focused on gross size and shape rather than fine detail. The two solid clavicle models are sent to the hospital via mail courier once completed. The model production process takes approximately 2 to 3 days from initial CT scan to obtaining the solid model. The total cost of each clavicle replica is under US$100.

### Selection of the anatomically precontoured locking plate

**Fig. 3:**
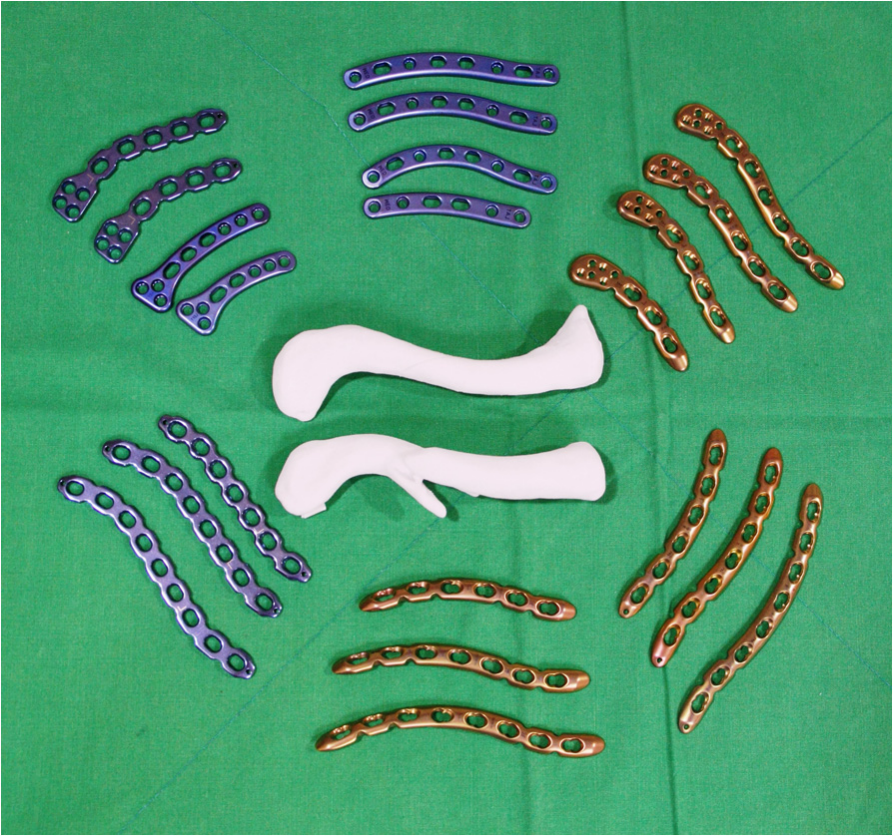
The real-size 3D-printed normal clavicle model is used as a template to select the plate, which best fits the model. All available plates are evaluated by a simulated attachment to the model

**Fig. 4:**
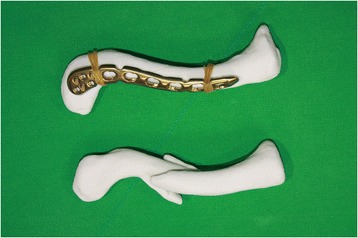
A plate that suitably fits the model is selected. It is important that at least three screws can be fixed to the lateral and the medial side of the fracture

There is a selection of anatomically designed precontoured locking plates for clavicle fractures available. Plates can be sourced in differing shapes and sizes and from a range of manufacturers. The real-size 3D-printed uninjured clavicle model is utilized as a template to select the plate, which best fits the model. A locking reconstruction plate can be molded to fit on the clavicle; however, we prefer not to bend or mold the plate but to select an anatomically contoured locking plate as bending or molding the plate may reduce fatigue strength of the plate which has the potential for plate breakage. All available plates are evaluated by a simulated attachment to the model (Fig. 3). The selected plate must suitably fit the clavicle model and provide a framework for the attachment of at least three screws on both the lateral and the medial side of the fracture (Fig. 4). Careful review of the radiographs, CT scan images, and the 3D-printed model of the fractured clavicle is required to select the proper position for plate fixation and to select which holes to use in the plate. Fluoroscopic images of the plate attached to the model are obtained to assist in proper plate positioning prior to the actual insertion. The plate and the 3D-printed model are sterilized prior to the surgery and the model is used intraoperatively as a reference for anatomic reduction of the fracture.

### Operative technique

**Fig. 5:**
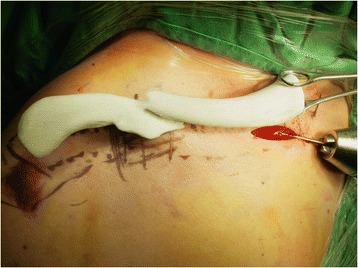
A small incision is made on the medial end of the fractured clavicle and a titanium elastic nail is inserted for the reduction of the fracture. The 3D-printed fractured clavicle model is used to assist the surgeon reduce the fracture by obtaining the fracture configuration intraoperatively

**Fig. 6:**
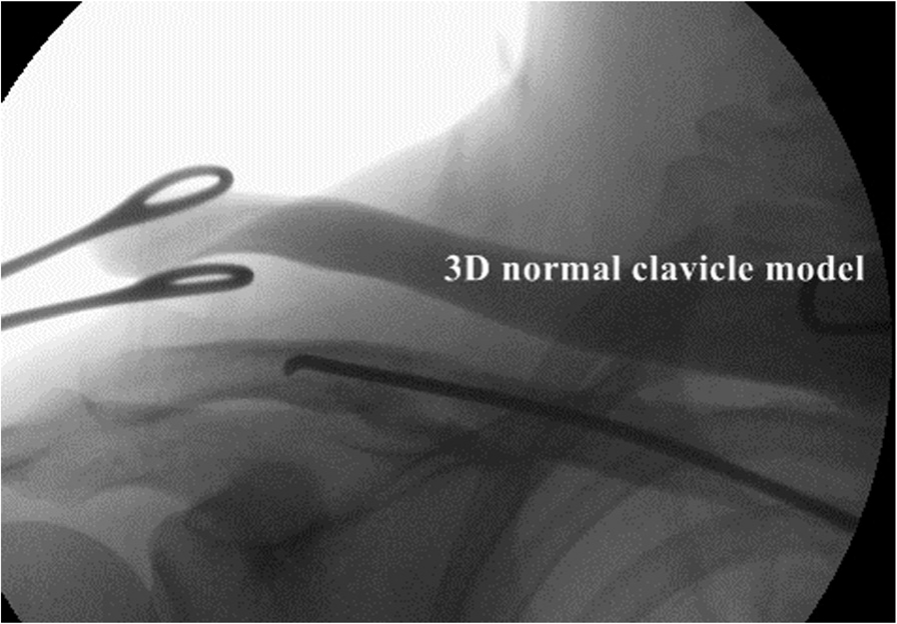
The real-size 3D-printed normal clavicle model is positioned over the fractured clavicle and the fracture reduction. The overall alignment and the length are verified under C-arm fluoroscopy by comparing it with the real**-**size model

**Fig. 7:**
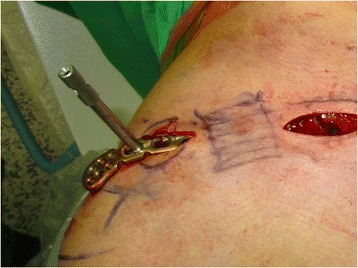
The precontoured plate is inserted underneath the subcutaneous tunnel over the periosteum without opening the fracture site

**Fig. 8:**
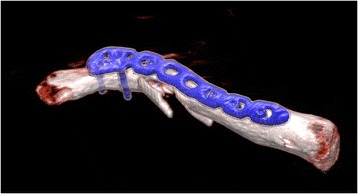
A minimum of three screws are fixed at either side of the fracture

The patient is placed in a beach chair position on a radiolucent table under general anesthesia. Before sterile draping, a C-arm is introduced parallel to the clavicle to ensure that adequate intraoperative fluoroscopic images can be obtained. After sterile draping, fluoroscopic images are taken with the plate attached to the 3D-printed model. This process assists in determining the proper position of the plate on the fractured clavicle during the insertion procedure. The fracture is reduced by insertion of a titanium elastic nail along the medullary canal of the clavicle under fluoroscopic guidance as described by Lee et al [8]. A small incision is made on the medial end of the fractured clavicle, and an entry hole is created by drilling the medical cortex of the clavicle. A titanium elastic nail is inserted for the reduction of the fracture. The 3-D printed fractured clavicle model is utilized to assist the surgeon with obtaining the fracture configuration and to reduce the fracture intraoperatively (Fig. 5). The 3D-printed normal clavicle model is used as a reference for the reduction (Fig. 6). The sterilized model is positioned over the fractured clavicle to allow for the fracture reduction, and the overall alignment and length to be assessed by C-arm fluoroscopy (Fig. 6). After satisfactory reduction, one small incision is made on the superior surface of the lateral segment. The previous incision made on the medial end is then extended to the anterosuperior surface of the medial segment for screw fixation on the plate. A subcutaneous tunnel is constructed from the lateral incision to the medial incision (or vice versa) on the clavicle by using the periosteal elevator or the edge of the plate without opening the fracture site. The precontoured plate is inserted underneath this subcutaneous tunnel over the periosteum without opening the fracture site (Fig. 7). The proper position of the plate is determined under C-arm fluoroscopy and by the aid of the fluoroscopic images taken previously with the plate attached to the 3D-printed model. Locking screws are placed through the lateral and medial small incisions, respectively. The nail inserted for the reduction is retrieved. A minimum of three screws are inserted either side of the fracture (Fig. 8). Postoperatively, patients are provided with an arm sling for 2 weeks. Pendulum and range of motion rehabilitation exercises are encouraged as tolerable.

Ethical standards were followed in the content and dissemination of the study.

## Results

Seven comminuted clavicle fractures treated with this technique achieved good bone union. The 3D-printed fractured clavicle model allowed the surgeon to observe and manipulate accurate anatomical replicas of the fractured bone to assist in fracture reduction. The 3D-printed uninjured clavicle model could be utilized as a template to select the anatomically precontoured locking plate which best fits the model. The plate could be inserted through a small incision and fixed with locking screws without exposing the fracture site.

## Discussion

The 3D printing technologies are common in product design industries, and their use is growing in all fields including medicine [12–17]. As the popularity of 3D printing is increasing, it is becoming financially feasible and accessible to use in orthopedic surgery [18, 19]. Use of 3D printing for acetabular fracture surgery has been reported, and its application to other fracture surgery is expected to increase as actual osseous anatomy can be reproduced that can help surgeons understand the characteristics of fractures [20, 21]. Although a bony surface structure can also be visualized by the 3D reconstructed CT images, it can only be visualized on the computer screen and the images have to be memorized for use during fracture reduction. 3D-printed fracture models can be utilized in the surgical field to assist surgeons with obtaining the correct fracture configuration during reduction. The uninjured side clavicle model printed as a mirror image can be utilized as a reference for anatomic reduction during fracture surgery. 3D printing is especially useful for non-extremity fractures, such as clavicle, acetabulum, and pelvis, as it is difficult to perform the C-arm manipulations required to obtain suitable fluoroscopic views for a 3D orientation of the fractured or uninjured sites. These views are critical as they provide surgeons with key images to use as a reference for anatomical reduction. Precontouring the plate or selecting the plate that will best fit the fractured bone is difficult without the 3D-printed model unless the fracture is exposed.

3D models can be invaluable for a precise preoperative plan as the plate can be attached to the 3D-printed fracture model prior to surgery. This allows the surgeon to select the correct screw holes to use or to perform a surgery on the 3D model prior to the real fracture surgery. The 3D models can also be utilized to educate residents and can enhance communication with patients.

The disadvantage of this technique is that considerable time and cost are required from the acquisition of the CT data to manufacturing and receiving the real-size 3D-printed model. However, the use and popularity of 3D printing in industry is growing at an exponential rate. This growth can be associated with lower printing costs and subsequently greater accessibility to printing technologies in the future.

Compared to open reduction for simple clavicle fractures, closed reduction requires increased fluoroscopic time and radiation exposure. During MIPO, it is sometimes difficult to reduce the fracture using a closed technique. Intramedullary nailing followed by plate fixation was introduced to make closed reduction easier [8]. However, this technique still requires longer operative time and increases the patient’s exposure to radiation, compared to open reduction. As CT scanning is not routinely used for simple clavicle fractures, obtaining CT scans for 3D printing in these cases increases radiation exposure. However, for comminuted clavicle fractures or fractures with poor skin conditions, for which open reduction could increase the risk of nonunion or wound complications, we believe our technique of using 3D printing for MIPO outweighs the risk of radiation exposure or prolonged operative time, compared to open procedures. CT scanning is helpful in these cases not only for 3D printing but also for obtaining the fracture configuration especially when MIPO is used for comminuted clavicle fractures. Compared to the standard technique for MIPO, our approach of using the 3D printed clavicle model to assist surgeons in obtaining the fracture configuration can make closed reduction easier and reduce fluoroscopy time, which lowers radiation exposure. However, our recommendation is only limited to MIPO used for comminuted fractures. For simple, displaced fractures of the clavicle, we believe open reduction and plate fixation should be the standard operative treatment.

## Conclusion

The 3D-printed fractured clavicle model allows the surgeon to observe and manipulate accurate anatomical replicas of the fractured bone to assist in fracture reduction prior to surgery. Using the mirror imaging technique, the uninjured clavicle model can be used as a template to select or pre-shape the anatomically designed locking plate for the minimally invasive plate osteosynthesis of the displaced comminuted midshaft clavicle fracture. The 3D-printed clavicle model can also be used intraoperatively as a reference for anatomic reduction.

### Consent

All authors certify that this study was approved by the Institutional Review Board of Hallym University Kangnam Sacred Heart Hospital and that patients' informed consent was obtained.
